# Clinical Observations and Occurrence of Complications following Heavy Silicone Oil Surgery

**DOI:** 10.1155/2014/706809

**Published:** 2014-04-14

**Authors:** Hendrik Schwarzer, Babac Mazinani, Niklas Plange, Matthias Fuest, Peter Walter, Gernot Roessler

**Affiliations:** Department of Ophthalmology, RWTH Aachen University, Pauwelsstraße 30, 52074 Aachen, Germany

## Abstract

*Purpose.* To demonstrate development and complications in heavy silicone oil (HSO) surgery in 100 eyes following primary vitreoretinal surgery. *Methods.* 100 eyes were included in this retrospective study that underwent vitreoretinal surgery using HSO as endotamponade. Indication diagnoses were retinal detachments (*n* = 76), complicated macular holes (MH) (*n* = 20), and others (*n* = 4). HSO removal was performed after a mean period of 20.2 ± 19.0 weeks. In 18 eyes with poor functional prognosis the silicone oil remained permanently for stabilisation. Overall follow-up time was 35.9 ± 51.8 weeks. *Results.* The mean IOP before HSO surgery was 13.3 ± 5.6 mmHg and raised to an average maximum of 23.3 ± 8.5 mmHg postoperatively and decreased to 13.7 ± 7.2 mmHg after removal. Secondary IOP raise due to emulsification of the silicone oil endotamponade was seen in 29 eyes after 7.8 ± 4.5 weeks. Other complications being observed with HSO installed were persistent corneal erosion (*n* = 3) and prolonged anterior chamber inflammation (*n* = 29). In 13 eyes recurrent retinal detachments occurred during followup. *Conclusions.* According to our analysis HSO surgery might deliver satisfying results in complicated cases of ophthalmological surgery. However, potential complications should always be taken into account when making the decision if to use and when to remove HSO in complicated retinal surgery.

## 1. Introduction

In vitreoretinal surgery long-term endotamponades have become a helpful alternative to the already widely used short-term endotamponades such as air, sulfur hexafluoride (SF6), and octafluoropropane (C3F8) in complicated cases of retinal detachments, recurrent retinal detachments, trauma surgery, and proliferative vitreoretinopathy (PVR) [1–4].

HSO has been designed to overcome the disadvantages of silicone oil and gas endotamponades because they are heavier than water endotamponade agents. Because of their increased density, they provide a good endotamponade of both the inferior and the posterior pole in normal head positioning, making postoperative face-down positioning no longer necessary in certain conditions [5–7]. Hence, especially in the treatment of retinal detachments with large inferior breaks or PVR the characteristics of heavier than water endotamponades may appear beneficial compared to other endotamponades [5, 7, 8].

Nevertheless several complications have been reported due to HSO surgery, such as prolonged intraocular inflammation and secondary IOP raise with a possible relation to emulsification of HSO [9–15]. This retrospective clinical study was established to determine the functional and anatomical outcome of heavier than water silicone endotamponade surgery in complicated cases with special focus on the main complications that may occur in the short- and long-term course of time after the operation.

## 2. Methods

The records of 100 patients and 100 eyes, respectively, which have undergone vitrectomy combined with HSO endotamponade between 2008 and 2011, were reviewed. All patients were treated at the Department of Ophthalmology of the RWTH Aachen University. In most cases the indication diagnosis for vitrectomy with HSO was proliferative vitreoretinopathy (PVR) and/or patients with complicated retinal detachments (*n* = 76) including 5 patients with retinal detachment secondary to open globe injury. Some patients received HSO surgery due to complicated MH (*n* = 20). MHs are considered complicated by the authors when one of the following apply: (a) a history of MH for 6 months or more; (b) after primary or even secondary retinal surgery, for example, when short-term endotamponades, such as SF6 or C3F8, were unsuccessful; or (c) whenever a larger central substantial defect was appreciated when the indication for the operation was established. Other indications were endophthalmitis (*n* = 2) and macular hemorrhage (2). Two different types of HSO were used, Oxane HD (Bausch & Lomb; *n* = 27) and Densiron 68 (FLUORON; *n* = 73), respectively.

In all patients 20 gauge standard system vitrectomy was performed. In cases of retinal detachment surgery HSO was installed in direct exchange with perfluorodecalin (PFD).

Complete ophthalmological examination was performed before and after treatment, and a database was created which included several parameters that were subsequently analysed.

Visual acuity was measured using decimal charts and converted into LogMAR units for statistical purposes. Nonnumeric values, such as light perception (LP), hand motion (HM), and count fingers (CF), were decimally described: LP = 0.001 (LogMAR 3.0), HM = 0.01 (LogMAR 2.0), and CF = 0.02 (LogMAR 1.7).

The intraocular pressure (IOP) was measured by standard Goldmann applanation tonometry. Slit lamp examinations and direct or indirect funduscopy were performed at first visit, before and after surgery, and at each visit throughout the follow-up time. All patients were examined 6 weeks after surgery and thereafter every 6–8 or 10–12 weeks, respectively, depending on occurrence of complications, such as intraocular inflammation or IOP rise.

IOP raise is defined by the authors as a difference of 8 mmHg or more between the IOP measured before primary HSO surgery and the IOP at the time of HSO-removal indication or any IOP above 24 mmHg after the primary surgery. Values greater than 21 mmHg are assumed to be ocular hypertensive values that are to be monitored and if necessary even to be treated. Thus taking it from a mean IOP of 13.3 ± 5.6 mmHg at baseline examination before HSO surgery, an elevation of 8 mmHg would drop into the boundaries of the ocular hypertension range. Moreover any IOP higher than 24 mmHg is supposed to be treated due to regularities of our clinic as it exceeds the upper range of ocular hypertension.

The mean follow-up time was 35.9 ± 51.8 weeks after last surgery.

For statistical purposes in the matter of the IOP comparison and development, a student* t*-test was performed using* IBM SPSS Statistics Standard*.

## 3. Results

In 82 of 100 eyes, HSO was removed after a mean period of 20.2 ± 19.0 weeks. In 18 eyes with poor functional prognosis, the silicone oil remained permanently for stabilisation at final visit.

At the time of HSO surgery indication the mean best corrected visual acuity (BCVA) was LogMAR 1.0 ± 0.8. At last follow-up examination after HSO removal the mean BCVA was 0.7 ± 0.7 which demostrates a mean BCVA improvement of 3 lines logMAR.

In 76 eyes, HSO was used as endotamponade following vitrectomy for treatment of retinal detachments due to inferior breaks with or without PVR. Out of those 76 eyes in 42 cases PVR reaction was seen. Following the European Vitreoretinal Society (EVRS) staging for PVR in 3 eyes stage A, in 8 eyes stage B, in 12 eyes stage C1, in 10 eyes stage C2, in 7 eyes stage C3, and in 2 eyes stage C4 could be observed at the time of HSO surgery indication. The primary success rate for this procedure was 82.9% (63 of 76 patients). 3 eyes (4.0%) showed a persistent retinal detachment under HSO endotamponade and 10 eyes (13.2%) a retinal redetachment occurred after removal of the endotamponade.

49 of all patients (49%) were phakic prior to first surgery out of which in 11 patients (22.4%) primary vitrectomy was combined with phacoemulsification. In the 38 remaining phakic patients cataract progression was observed in 22 eyes (57.9%) requiring cataract surgery simultaneously to HSO removal.

**Figure 1:**
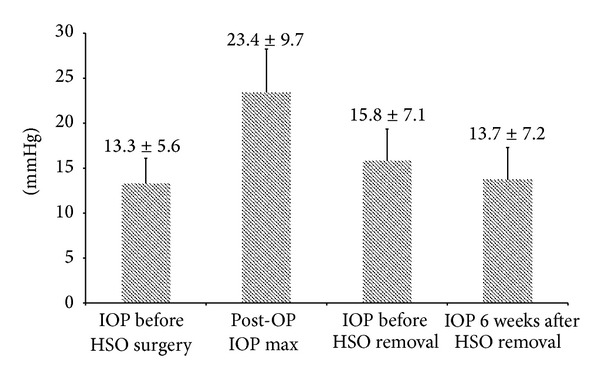
IOP development from pre-HSO surgery through 6 weeks after HSO removal.

At the time of HSO surgery indication the mean IOP was 13.3 ± 5.6 mmHg. With HSO installed the IOP rose to an average maximum of 23.3 ± 8.5 mmHg within the first few days postoperatively and could be lowered to a mean IOP of 15 ± 5.4 mmHg by using 1 to 4 different topical antiglaucomatous agents. In 15 patients (15%) the IOP rose above 30 mmHg within the first few days postoperatively. Within 7.7 ± 4.5 weeks after primary HSO surgery the IOP rose up to maximum values of 56 mmHg and a mean IOP of 23.4 ± 9.7 mmHg within 7.8 ± 4.5 weeks postoperatively. At 6 weeks postoperatively a mean IOP of 15.6 ± 8.1 mmHg was seen and still was significantly higher than preoperatively (*P* = 0.007). 15 patients needed 1 or 2 different topical antiglaucomatous agents to keep the IOP stable and 1 patient needed more than 2 different agents. At resurgery indication a mean IOP of 17.7 ± 8.5 mmHg was seen. Meanwhile 20 patients needed topical antiglaucomatous therapy out of which 9 patients needed 1 agent, 7 patients needed 2 agents, and 4 patients needed 3 or 4 different agents. In 8 patients extra systemic sulphonamides were needed to control the IOP until HSO-removal surgery. At the date of surgery the preoperative mean IOP was 15.8 ± 7.1 mmHg. After removal of HSO the IOP decreased to 13.7 ± 7.2 mmHg 6 weeks postoperatively (*P* = 0.018). Only 8 patients still needed topical antiglaucomatous therapy at that time out of which only 3 needed more than one agent. An overview of the IOP development throughout the whole treatment is given in Figure 1.

**Figure 2:**
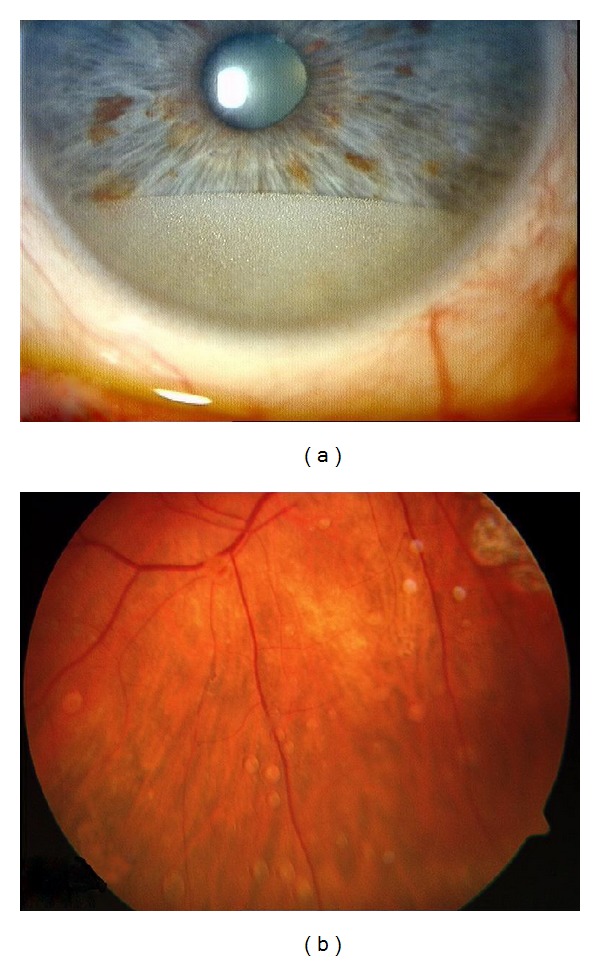
(a) Emulsification of HSO in the anterior chamber. (b) Emulsificated HSO adhesive to the retina.

In 37 patients emulsification of the HSO was observed by slit lamp examination, gonioscopy, or indirect funduscopy as displayed in Figures 2(a) and 2(b), respectively.

In 22 eyes secondary IOP rise was seen after a mean period of 7.8 ± 4.5 weeks. In all of these 22 cases emulsification of the silicone oil endotamponade was observed. In 15 more cases emulsification without IOP rise could be seen.

**Figure 3:**
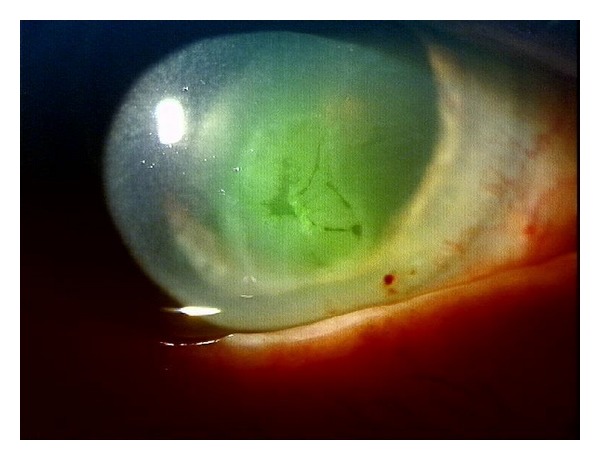
Example of a persistent corneal erosion after HSO endotamponade surgery.

Other complications being observed with HSO installed were persistent corneal erosion (*n* = 3) (Figure 3) and prolonged anterior chamber inflammation (*n* = 29) out of which the majority (*n* = 20) was recurrent after HSO removal within the first 6 weeks postoperatively. In the residual 9 patients that still had anterior chamber inflammatory signs at the 6 week follow-up visit, the inflammation was recurrent within a few more weeks using topical steroids.

In 2 cases after HSO removal a cystoid macular edema occurred that was persistent throughout all follow-up visits and could neither be controlled with topical or intravitreal steroids nor with intravitreal bevacizumab.

## 4. Discussion

The most common indication for the use of heavier than water endotamponades is the use for the treatment of retinal detachments with inferior pathologies. In previous studies the primary success rate has been determined between 54% and 89% [5–7, 9, 16–18]. The high range in these rates may be explained by heterogenous preoperative retinal findings that seem to be relevant especially in smaller case series. However, in our study the primary success rate of approximately 83% was reached in a comparatively large cohort with, in our opinion, a representative case mixture. This may confirm the effectiveness of HSO in these indications.

In the literature the most frequently reported complication in the use of HSO is the progression of lens opacities with rates from 38% up to 100% [6, 7, 12]. These observations reveal certain limitations especially in retrospective studies, such as in this study, as there is little information about the preoperative cataract grade. On the other hand it is not clear whether cataract progression is primarily caused by the endotamponade or by the vitrectomy itself, which even if there is no documentation about intraoperative lens damage represents a risk factor for postoperative progression of lens opacities [19]. Finally, our results do not allow any statement about the progression in relation to endotamponade duration. However, while the combined surgery of cataract and HSO extraction is a common and feasible procedure, cataract formation represents an acceptable complication.

The two major issues in the use of heavier than water endotamponades seem to be secondary IOP rise after HSO surgery that in some cases even persists after HSO removal and a prolonged intraocular inflammation that seems to be induced by the HSO. In regard to the elevation of IOP we detected two peaks, one immediately after surgery, which could be controlled by conservative local or systemic antiglaucomatous therapy. There are several possible reasons for an early increase of IOP including inflammation, application of laser photocoagulation, the use of encircling bands, a pupillary block, or migration of silicone oil into the anterior chamber [20–22].

Wong et al. [23] described a postoperative early increase of IOP that was significant higher at the first postoperative day compared to another group of patients that underwent conventional silicone oil surgery. According to their results at day one postoperatively in 9 out of 71 patients (12.7%) the IOP rose above 30 mmHg after HSO surgery, while according to our results in 15 out of 100 patients (15%) the IOP rose above 30 mmHg within the first few days postoperatively. Wong et al. also state in their work that after 4 weeks the mean IOP deteriorated to 18.8 ± 9.4 mmHg. In our clinic the first planned visit after hospital discharge was after 6 weeks. After that period the mean IOP decreased to a value of 15.6 ± 8.1 mmHg. Our results are comparable to findings reported by Wong et al. in which IOP demonstrated an early rise postoperatively followed by a subsequent IOP decrease seen after 4 weeks.

In our patients we observed that inflammation and intraoperative laser might be the most common reasons for this early hypertension due to the absence of scleral buckling, pupillary block, or migrated silicone oil in the majority of the eyes.

In all cases where a second IOP elevation occurred after a followup of six weeks we found emulsification of HSO, which may have reduced the aqueous humour outflow. In the literature the rate of emulsification is indicated with rates between 5% and 18.5% [9, 18, 24]. These studies have in common that smaller amounts of cases have been investigated in each study and the mean endotamponade duration was shorter compared to our study. This may explain the higher rate of emulsification we found in our case series. However, removal of HSO including aspiration of emulsified bubbles out of the anterior chamber transferred IOP values to normal levels in most of the cases without requiring further antiglaucomatous therapy.

Romano et al. [25] described the development of a hyperviscous solution that could be described as “sticky oil” being generated by exchanging perfluorocarbon liquids (PFCL) such as PFD directly with HSO instead of air and HSO thereafter intraoperatively. In our opinion this could be a reasonable explanation for the relatively high complication rate, since our treatment regime regularly includes the direct exchange of PFCL and HSO.

The authors' decision to remove the HSO endotamponade strongly depended on the anatomical stability, functional outcome to be expected, and/or the occurrence of intraocular inflammation of the individual patient's eye, which explains the relative wide standard deviation (SD) of the mean residence time of the HSO. The wide SD in the follow-up time as well as in the IOP measured at any time after the HSO operation is explained by the fact that this work was a retrospective clinical study and some patients had their last visit 6–8 weeks after HSO removal whereas other patients were monitored for several more months after the last operation due to a more complicated development and eventually in some patients the silicone oil remained permanently for stabilisation, due to poor functional prognosis or a probable development of hypotonia following trauma.

In two patients we observed the occurrence of a cystoid macular edema during followup after HSO removal. Neither a treatment with topical and systemic steroids nor intravitreal injections with triamcinolone-acetonide or bevacizumab showed any effect on these findings. To our knowledge this is the first report on chronic macular edema following HSO surgery and stands in contrast to all other cases of persistent intraocular inflammation in our study, which were treated successfully during followup after HSO removal.

We suppose that a pathogenetic factor could be a proinflammatory influence of the HSO that seems to be persistent even after HSO removal. This assumption, of course, cannot be proved with the data of this study, since this complication was observed in only two patients, so a larger cohort of patients needs to be observed in future investigations.

In most studies HSO was removed within three months and by now its feasibility as a long-term endotamponade could not definitely be proven [4–7, 9]. In fact, in our study inflammation or IOP elevation, if not sufficiently controllable, accelerated the decision to remove HSO. However, in more than two-third of all patients from the time of having HSO installed no major problems occurred, allowing the suggestion to remove HSO in regard to the anatomical situation of the retina alone. Moreover, in some cases, especially in eyes that needed antiproliferative HSO surgery after severe trauma, the HSO seems to stabilise the anatomical constitution and restrain the eye from IOP drop, persistent hypotonia, and phthisis by leaving HSO as permanent endotamponade installed.

In conclusion, in our study, as no alarming complications occurred in the majority of cases, safety of HSO endotamponade could be demonstrated. However, eyes carrying HSO need frequent follow-up examinations as the appearance of inflammations or IOP elevations could influence the decision of when to remove the endotamponade.
